# A Novel Record of Brown Hyaena ( *Parahyaena brunnea* ) in Zinave National Park, Mozambique

**DOI:** 10.1002/ece3.72473

**Published:** 2025-11-11

**Authors:** Osvaldo J. Abrao, Olivia Sievert, Marnus Roodbol, Lizanne Roxburgh, Bernard van Lent, Antonio Abacar, Ana Gledis da Conceição, Gold Chinder, Isildo de N. Nganhane, Samantha K. Nicholson

**Affiliations:** ^1^ Peace Parks Foundation, Zinave National Park Inhambane Province Mozambique; ^2^ The Endangered Wildlife Trust Johannesburg South Africa; ^3^ National Administration of Conservation Areas Maputo Mozambique; ^4^ Faculty of Natural Sciences Lúrio University Pemba Mozambique; ^5^ Wildlife Conservation Research Unit Oxford University Oxford UK

**Keywords:** camera trapping, carnivore, distribution, ecological restoration, hyaenidae, monitoring

## Abstract

Globally, large carnivores face significant threats and have lost substantial portions of their historical range. The Brown Hyaena ( *Parahyaena brunnea* ), one of four Hyaenidae species, has a global population estimated at fewer than 10,000 individuals and the species is of conservation concern. Its population size in Mozambique remains unknown. Zinave National Park (Zinave) in Mozambique is undergoing recovery following the impacts of a prolonged civil war, which severely depleted its wildlife populations. Recently, however, the park has seen the return of large carnivores, both naturally (Spotted Hyaena; *Crocuta crocuta* , Leopard; *Panthera pardus* , and Lion; *Panthera leo* ) and reintroduced (Spotted Hyaena and Leopard) inside the sanctuary. During September 2023 to September 2024, through the establishment of a permanent camera trap grid and the periodic placement of camera traps on carcasses to monitor scavenger activity, the first images of Brown Hyaenas were recorded in Zinave. This provides evidence of their presence in an area not previously considered within the species extant range. This study highlights the importance of long‐term biodiversity monitoring, both inside and outside protected areas, using complementary methods such as active search efforts and camera trapping. These approaches are critical for documenting rare and cryptic species, species range shifts and generating essential data to guide effective conservation strategies.

## Abstract 2

This paper describes a novel record of Brown Hyaena observations in Zinave National Park (Mozambique) which is not currently included in the extant range.

## 1 Introduction

Zinave National Park (hereafter Zinave), in the south of Mozambique, is a critical protected area as it is integral to regional connectivity and is contiguous with Banhine and Limpopo National Parks (Mozambique) and Gonarezhou National Park (Zimbabwe) and two wildlife management areas (Coutada 4 and Coutada 5; Mozambique). Coutada 4 and 5 are located to the north‐east of Zinave and are hunting concessions with limited formal management (DNAC 2009). Zinave has experienced severe declines in its large and medium‐sized mammal populations due to a combination of factors, including the 16‐year civil war in Mozambique, recurrent droughts, and a critical lack of management capacity (Hanks 1997; ZNP 2010). During the civil war, all large carnivores became locally extirpated due to unnatural anthropogenic pressures (Loft 2018). Several carnivore species historically occurred in Zinave including Lion ( *Panthera leo* ), Leopard ( *Panthera pardus* ), Cheetah ( *Acinonyx jubatus* ), Spotted Hyaena ( *Crocuta crocuta* ), Black‐backed Jackal (*Lupulella mesomelas*) and Wild Dogs ( *Lycaon pictus* ) (Dalquest 1965; Tinley et al. 1976). In 2015, a new era began with the signing of a co‐management agreement between the Peace Park Foundation and the Government of Mozambique. This agreement led to significant improvements in financial input and subsequent management capacity and ecosystem rehabilitation, including the hiring of additional rangers, the acquisition of new equipment, and reintroduction of over 2000 herbivores into the park (PPF 2020). In 2020 four Spotted Hyaenas were reintroduced into Zinave followed by two Leopards in 2021. In late 2021, the first non‐reintroduced Lion was sighted in Zinave, and in 2023, three more Lions and one non‐reintroduced Leopard were observed (Abrao 2024). As of 2025, it is estimated that there are seven Lions in Zinave based on observational records and individual identification (Abrao 2024).

The Brown Hyaena ( *Parahyaena brunnea* ) occurs throughout the arid zone of southern Africa, occurring in parts of Angola, across Namibia, Botswana, South Africa and Zimbabwe (Wiesel 2015; Welch et al. 2016). The species is listed as Near Threatened, with a global population estimated to be less than 10,000 mature individuals (Wiesel 2015). The extent of distribution of Brown Hyaena in Mozambique is currently unknown (Wiesel 2015; Yarnell et al. 2016). There are no historic records of Brown Hyaena in Zinave, and here, we document the first confirmed record of the species.

## 2 Methods

### 2.1 Study Area

**FIGURE 1:**
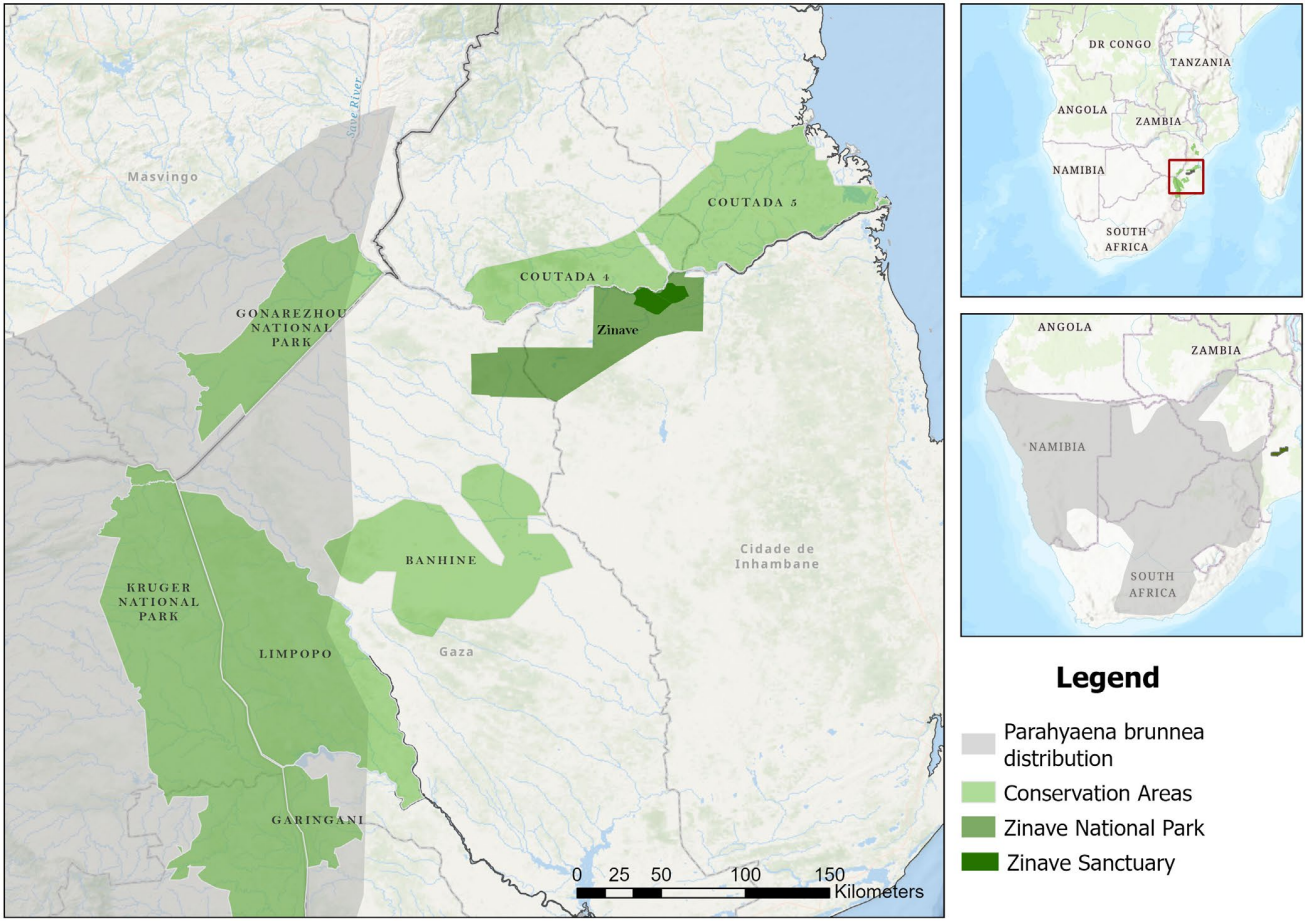
Map showing Zinave National Park in proximity to Greater Limpopo Transfrontier Conservation Areas and the most recent distribution for the Brown Hyaena.

This study was conducted in Zinave, Mozambique (−21.577 S; 33.527 E; Figure 1). The park covers an area of 4091 km^2^ and is part of the Greater Limpopo Transfrontier Conservation Area (GLTFCA). Zinave occurs in the mopane and miombo woodland interface in the Sudano‐Zambezia biogeographical region. Its main landscapes include *Acacia nigrescens* forest, sandveld, miombo, mopane, Save river channel, Save riverine forest and wetland (Stalmans and Peel 2010) and represents an important transition zone between tropical humid and arid tropical environments (Tinley et al. 1976). The region's climate ranges from tropical to subtropical, with average monthly temperatures exceeding 18°C, even during the cooler winter months of August and September (Stalmans and Peel 2010). Rainfall is higher during the summer months, peaking in February, and lowest during the winter. Annual precipitation averages vary between 690 mm in the northeast and 570 mm in the far west. Historically, the Zinave landscape supported diverse populations of large mammals. However, colonial hunting, followed by a prolonged civil war during which wildlife was hunted for food, left the area significantly depleted of these animals (ZNP 2010; Gray and Crist 2023).

**FIGURE 2:**
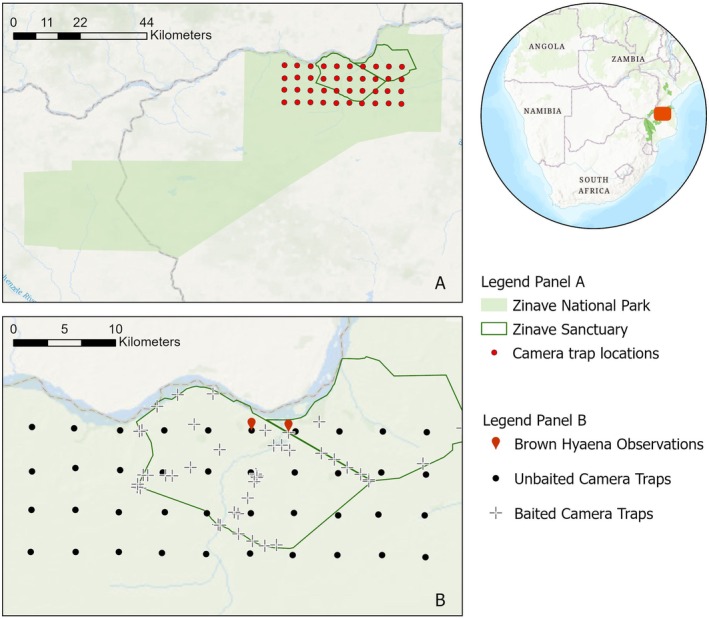
Maps showing the camera trap grid of baited and unbaited traps and Brown Hyaena observation. Panel A: The red dots indicate only the location of the permanent camera trap grid. Panel B: The black dots indicate the location of permanent and unbaited camera trap grid, the crosses indicate the baited camera traps, and the red dots indicate the cameras where the Brown hyaena was recorded.

Zinave's herbivore guild includes Buffalo ( *Syncerus caffer* ), Elephant ( *Loxodonta africana* ), Giraffe ( *Giraffa giraffa* ), Impala ( *Aepyceros melampus* ), Blue Wildebeest ( *Connochaetes taurinus* ), Zebra ( *Equus quagga* ), Warthog ( *Phacochoerus africanus* ), Waterbuck (*Kobus elipsiprymnus*), Sable ( *Hippotragus niger* ), Reedbuck ( *Redunca arundinum* ), Eland (*Tragelaphus oryx*), Black Rhinoceros ( *Diceros bicornis* ) and White Rhinoceros ( *Ceratotherium simum* ) that were all reintroduced between 2016 and 2023. In addition to herbivore species, Ostriches ( *Struthio camelus* ) were also reintroduced into Zinave. Zinave management has established a 6 km^2^ wildlife sanctuary (Figure 2) which has gradually expanded and now covers around 35 km^2^. All reintroduced individuals were released within the sanctuary; however, they are able to move freely throughout Zinave which is an unfenced park.

### 2.2 Camera Trap Grid

A long‐term camera trap grid with 40 cameras was installed in an area of 432 km^2^ covering several habitats inside and outside the sanctuary within Zinave. The camera traps in the grid were spaced 4 km apart. Two models of cameras were used: (i) Cuddeback with white Xenon flash (Change Color Model 1279; Cuddeback, Green Bay, WI, USA) which were installed inside the sanctuary; and (ii) Uovision Glory 4G LTE infrared camera (Uovision, Shenzhen, China) which were installed outside the sanctuary. All camera traps within the long‐term camera trap grid were set up on 30 June 2023 and were active from 1 July 2023 at 00:00. The collection date was set as 30 June 2024 at 23:59.

All cameras were placed preferably on the tree, 1 m above the ground to obtain a greater range, especially in areas with relatively tall grass (Gaynor et al. 2021), protected by a metal box and with a slightly inclined angle. Strategically, game trails were often chosen within the grid to maximize the success of captures, and we avoided any position that would have prolonged sun exposure to prevent false shots (Gaynor et al. 2021). Cameras were programmed to operate in photo mode, in normal‐to‐medium sensitivity, capturing three pictures at once, with 30 s intervals between triggers.

### 2.3 Opportunistic Carcass Monitoring

To identify and monitor the scavenger guild present in Zinave, a camera trap was opportunistically installed at each carcass that was found to have resulted from natural mortality during routine monitoring and remained in place until the carcass had completely decomposed or disappeared. Opportunistic carcass monitoring took place between May 2023 and September 2024.

### 2.4 Data Collection, Processing and Identification

Species identification was conducted manually by the Endangered Wildlife Trust's research team.

## 3 Results

During the survey, 13,643 photos were captured from the 40 unbaited camera traps in the long‐term camera trap grid, over a period of a year, capturing 37 species. From the 44 opportunistic carcass monitoring events, 3718 photos were captured from baited camera traps identifying 11 species of scavenging herbivores and five species of scavenging birds (Table 1).

**TABLE 1:** Species captured on the baited camera traps May 2023 and September 2024.

Species of carcass	Number of carcasses placed	Mammal scavengers captured	Bird scavenger captured
Elephant	1	Spotted Hyaena, Civet ( *Civettictis civetta* ) Bushpig ( *Potamochoerus larvatus* )	
Buffalo	5	Lion, Spotted Hyaena, Civet	White‐backed Vulture ( *Gyps africanus* ), Marabou Stork (*Leptoptilos crumenifer*)
Zebra	1	Lion, Side‐striped Jackal (*Lupulella adusta*), Civet, Honey Badger (*Melifora capensis*)	
Blue Wildebeest	5	Lion, Spotted Hyaena, Civet	White‐backed Vulture
Waterbuck	7	Spotted Hyaena, Civet, Crocodile ( *Crocodylus niloticus* )	White‐backed Vulture, White‐headed vulture
Kudu	3	Spotted Hyaena, Civet, Bushpig, Honey Badger	White‐backed Vulture, Ground Hornbill ( *Bucorvus leadbeateri* )
Nyala	4	Lion, Spotted Hyaena, Civet, Caracal, Bushpig	Ground Hornbill, White‐headed vulture
Impala	6	Lion, Spotted Hyaena, Brown Hyaena, Serval, Caracal, Genet	White‐backed Vulture, Marabou Stork, Bateleur
Bushbuck	3	Spotted Hyaena	White‐backed Vulture
Warthog	8	Spotted Hyaena, Civet	White‐backed Vulture
Bushpig	1	Spotted Hyaena	White‐backed Vulture
Total	44	9	5

**FIGURE 3:**
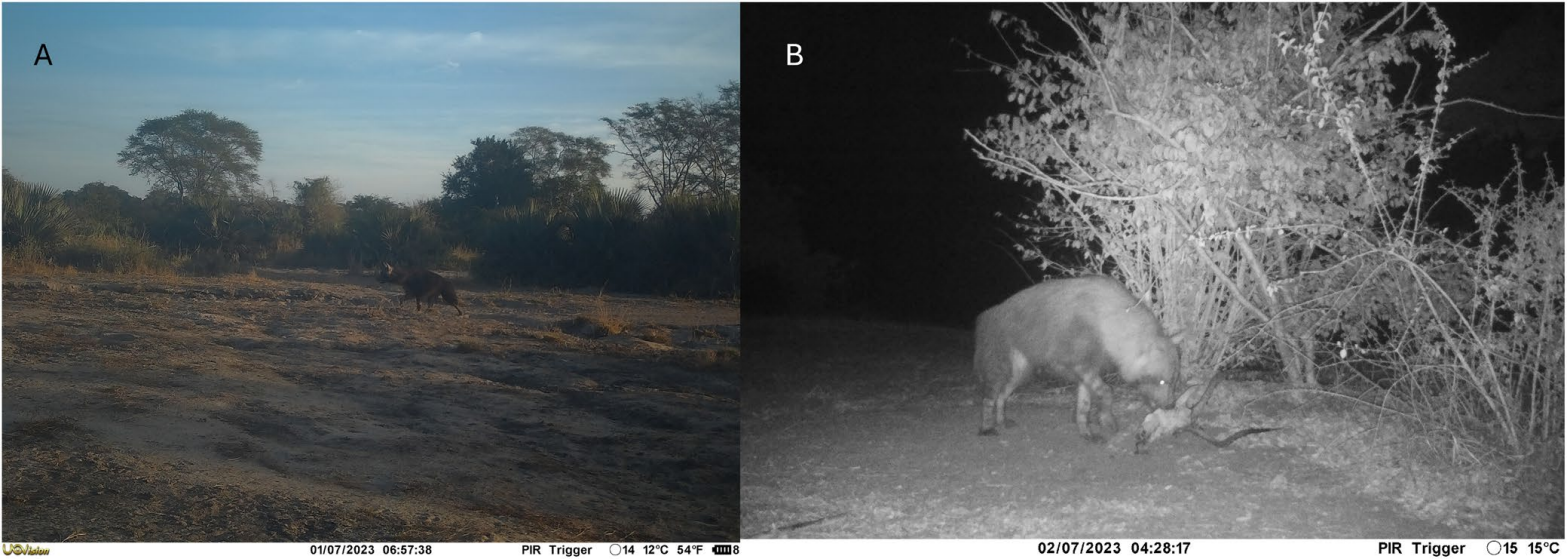
Camera trap images of the first Brown Hyaena observed in Zinave National Park, Mozambique. Panel A: The first documented images of the species in the park within the camera trap grid. Panel B: Brown Hyaena image captured on a baited camera trap.

During the study, one camera trap in the long‐term camera trap grid and another camera trap station from our carcass monitoring captured images of Brown Hyaena (Figure 2) in Zinave. These captures took place on the 1st and 2nd of July 2023, corresponding to a single individual over a 24‐h period. Individual identification was based on individual markings and the proximity of each observation in location (3 km distance between observations) and time (within 24‐h). The Brown Hyaena was recorded walking (Figure 3A) and feeding from an impala carcass (Figure 3B) and during the event nine images were recorded. All photographic captures were recorded early morning between 04:28 a.m. and 06:57 a.m. Detections occurred in the Zinave pan floodplain and within an open savannah a few kilometers from the Zinave pan, early dry season.

## 4 Discussion

Long‐term biodiversity surveys are essential for detecting rare, cryptic and low‐density large‐medium mammal species when studying population dynamics, recovery and range shifts. Here, we report the first documented presence of the rarest Hyaena species, the Brown Hyaena, in Zinave which was confirmed through camera trapping. This discovery is a result of efforts to establish a wide and permanent camera trap grid across the park. Camera traps, due to their non‐invasive and silent nature, are effective for recording elusive, transient or rare species that are difficult to observe through traditional methods (Balme et al. 2009; Thorn et al. 2009; Edwards et al. 2020; Stratford et al. 2020; Sievert et al. 2023). Since the Brown Hyaena is a scavenger (Mills 1990), the growth of herbivore populations and the consequent return of Lions may have increased the availability of carrion in Zinave, thus increasing food availability for Brown Hyaenas (Bashant et al. 2020). Lack of food availability (Watts and Holekamp 2007), in addition to habitat reduction and fragmentation (Crooks et al. 2011; Schipper et al. 2008), is one of the main threats to Brown Hyaenas.

Due to the minimal ecological monitoring taking place prior to 2015, it is possible that Brown Hyaenas previously existed in Zinave and managed to remain in the areas adjacent to the park undetected. Brown Hyaenas have been shown to tolerate some level of land use change (Maude 2005; Pegg 2015; Rich et al. 2017), which is why a significant proportion of the population is found in unprotected areas (Kent and Hill 2013), although in low densities (Richmond‐Coggan 2014). Where there is a lack of carrion, as may have happened after the decline of herbivores and other carnivores in Zinave, Brown Hyaenas may have survived by relying on various food alternatives such as plant matter, fruits, invertebrates, reptiles and ostrich eggs (Mills and Mills 1978; Mills 1990). On the other hand, it is possible that the Brown Hyaena is a disperser that arrived from known range areas such as Limpopo or Gonarezhou National Parks (Williams et al. 2021). Given that this was the only capture of a Brown Hyaena over the yearlong survey and that no further reports of the species have been made, this is very likely. The minimal estimated distance from the area the Brown Hyaena was recorded in Zinave to known ranges in Banhine (130 km), Gonarezhou (180 km), Limpopo (250 km) and Kruger (303 km) National Parks.

The Brown Hyaena is known for its preference for arid and semi‐arid regions, including deserts and semi‐deserts (Adenekan, n.d.; Mills and Mills 1982). However, the Brown Hyaena is flexible in its habitat use at a landscape scale, from open grasslands of the riverbed or around the pans to open savannas or woodlands—all of which are associated with large carnivore kills and subsequent scavenging opportunities (Welch et al. 2016). In Zinave, the Brown Hyaena was observed near the lagoon and in the savannah, and the climate conditions are also somewhat similar, since Zinave is dominated by semi‐arid savannas (Bento et al. 2024).

As Brown Hyaenas are a typically uncommon component of predator guilds, and not studied as well as other large carnivores (Strampelli et al. 2022), it is especially important to document all records of their presence and activity to inform future ecological and conservation assessments. The previous distribution described for Brown Hyaena in Mozambique included an area of the Limpopo National Park (Wiesel 2015). We suggest this extant range be extended to encompass Zinave and the Save River, which forms the northern boundary of the park. In addition, we recommend continued monitoring of this area to determine how frequent this species is observed, especially as we believe this was just one individual. Over the last several years, carnivore species have returned to Zinave, demonstrating the restoration potential of the area. However, the current size of the population of Brown Hyaenas in the park and in Mozambique is still unknown due to gaps in biodiversity surveys. This is a challenge faced across Africa due to numerous obstacles that limit comprehensive sampling efforts (Nganhane and Farooq 2024). With the growth of herbivore populations in Zinave and a consequent increase in the amount of carrion, Zinave has the potential to become an important area for carnivores, including the Brown Hyaena. Due to its location, Zinave is in a unique position to have dispersing carnivores naturally recolonize the area, as seen by the recent Lion, Spotted Hyaena, and Leopard records. This highlights the important role of wildlife corridors in connecting protected areas across the landscape, and underscores Zinave's potential to become a stronghold for carnivores, provided that conservation efforts continue. The detection of a Brown Hyaena in Zinave, where carnivore numbers remain relatively low, is ecologically significant as it indicates early signs of trophic recovery and scavenger guild re‐establishment following decades of collapse. As an obligate scavenger, the species can contribute to nutrient cycling and carcass removal, thereby enhancing ecosystem function and potentially influencing disease dynamics. Continued recolonization (assisted or natural) by species such as the Brown Hyaena would therefore signal progress towards a more balanced and functional carnivore community in Zinave, reflecting broader ecological restoration success within the park.

## Author Contributions

**Osvaldo J. Abrao:** conceptualization (lead), data curation (lead), formal analysis (lead), investigation (lead), methodology (lead), project administration (lead), resources (lead), software (lead), supervision (supporting), validation (lead), visualization (lead), writing – original draft (lead), writing – review and editing (lead). **Olivia Sievert:** conceptualization (supporting), writing – original draft (supporting), writing – review and editing (supporting). **Marnus Roodbol:** writing – review and editing (supporting). **Lizanne Roxburgh:** conceptualization (supporting), writing – review and editing (supporting). **Bernard van Lent:** writing – review and editing (supporting). **Antonio Abacar:** writing – review and editing (supporting). **Ana Gledis da Conceição:** writing – review and editing (supporting). **Gold Chinder:** methodology (supporting), writing – review and editing (supporting). **Isildo de N. Nganhane:** conceptualization (supporting), writing – original draft (supporting), writing – review and editing (supporting). **Samantha K. Nicholson:** conceptualization (supporting), supervision (lead), writing – original draft (supporting), writing – review and editing (supporting).

## Conflicts of Interest

The authors declare no conflicts of interest.

## Supporting information


**Data S1:** Location of Camera traps that detected the presence of Brown Hyaena in Zinave.

## Data Availability

All the required data is uploaded as Supporting Information.
